# Supplementary material for: Transcriptome Sequencing and Metabolite Analysis Revealed the Single and Combined Effects of Microplastics and Di-(2-ethylhexyl) Phthalate on Mouse Liver
Source: Int J Mol Sci. 2025 May 21;26(10):4943. doi: 10.3390/ijms26104943 (PMC12112587; doi:10.3390/ijms26104943)
Supplement: Supplementary file 1 [file ijms-26-04943-s001.zip › ijms-3628002-supplementary.pdf]

Table S1. Antibody dilution details.

Antibody	Dilution ratio	Resource	Catalog
IL-6	1：800	Wanleibio, China	WL02841
TNF-α	1：800	Wanleibio, China	WL01581
Bax	1：800	Wanleibio, China	WL01637
Bcl-2	1：500	Wanleibio, China	WL01556
Caspase 3	1：800	Wanleibio, China	WL04004
Caspase 8	1：800	Wanleibio, China	WL03426
Caspase 9	1：800	Wanleibio, China	WL03421
AKT	1：1000	ABclonal, China	A22412
PI3K	1：1000	ABclonal, China	A27717
GAPDH	1：100000	ABclonal, China	A19056

Table S2. The primers used in this study.

Gene	Primer sequence （5'-3'）
IL-6 (NM_001314054.1)	Forward 5'- CCTCTCTGCAAGAGACTTCC - 3'
	Reverse 5'- GTTGGGAGTGGTATCCTCTG - 3'
TNF-α (NM_001278601.1)	Forward 5'- CCCTCACACTCACAAACCAC - 3'
	Reverse 5'- AAGGTACAACCCATCGGC- 3'
Bax (NM_001411994.1)	Forward 5'- AAGGCCTCCTCTCCTACTTC - 3'
	Reverse 5'- CTCAGCCCATCTTCTTCCAG - 3'
Bcl-2 (NM_009741.5)	Forward 5'- CCTTCTTTGAGTTCGGTGGG - 3'
	Reverse 5'- AAACAGAGGTCGCATGCTGG - 3'
Caspase 3 (NM_001284409.1)	Forward 5'- GGGAGCTTGGAACGGTACG - 3'
	Reverse 5'- CCAGAGTCCACTGACTTGCT - 3'
Caspase 8 (NM_001080126.2)	Forward 5'- CCTCCTCCTATGTCCTGTCT - 3'
	Reverse 5'- CTTCTCTGTCTGGGGTCTGT - 3'
Caspase 9 (NM_001277932.2)	Forward 5'- CACCTTCCCAGGTTGCCAAT - 3'
	Reverse 5'- CCATGAGAGCTTCGGAGAGA - 3'
AKT (NM_001165894.2)	Forward 5'- GCCGCCTGATCAAGTTCT - 3'
	Reverse 5'- GTTCAGATGATCCATGCGGG - 3'
PI3K (NM_001024955.2)	Forward 5'- GCGTGACATGTAGGCTCTCAG - 3'
	Reverse 5'- CTATACGGCCCGCACTGTAA - 3'
β-actin (NM_007393.5)	Forward 5'- GCAGGAGTACGATGAGTCC - 3'
	Reverse 5'- CAGCTCAGTAACAGTCCGC - 3'

Table S3. Sequencing raw data statistics.

Sample	Raw reads	Raw bases	Clean reads	Clean bases	Error rate(%)	Q20(%)	Q30(%)	GC content(%)
Con1	47685704	7200541304	47361738	7112206951	0.0238	98.49	95.37	49.52
Con2	44924742	6783636042	44616688	6708017230	0.024	98.4	95.15	50.16
Con3	45856592	6924345392	45515688	6815270365	0.0239	98.45	95.27	50.08
MPs1	46251338	6983952038	45879538	6894715321	0.0241	98.38	95.12	50.23
MPs2	43931550	6633664050	43558170	6532854344	0.0238	98.48	95.36	50.38
MPs3	55876990	8437425490	55305126	8308201613	0.0238	98.49	95.39	49.61
DEHP1	53121980	8021418980	52744476	7933004467	0.0239	98.45	95.3	50.77
DEHP2	42958038	6486663738	42591388	6402492885	0.0241	98.37	95.05	50.32
DEHP3	45792288	6914635488	45408678	6819909001	0.0238	98.47	95.35	49.56
MPs_DEHP1	46030712	6950637512	45738822	6866365350	0.024	98.41	95.16	49.85
MPs_DEHP2	49270192	7439798992	48841006	7329211439	0.0239	98.45	95.27	48.83
MPs_DEHP3	44797302	6764392602	44437298	6649147029	0.0236	98.6	95.62	49.78

Table S4. Comparison of sequencing raw data.

Sample	Total reads	Total mapped	Multiple mapped	Unique mapped
Con1	47361738	45749602(96.6%)	3813709(8.05%)	41935893(88.54%)
Con2	44616688	43100259(96.6%)	3618497(8.11%)	39481762(88.49%)
Con3	45515688	44041541(96.76%)	3794564(8.34%)	40246977(88.42%)
MPs1	45879538	44301180(96.56%)	3942899(8.59%)	40358281(87.97%)
MPs2	43558170	42165686(96.8%)	3610182(8.29%)	38555504(88.51%)
MPs3	55305126	53699927(97.1%)	5000107(9.04%)	48699820(88.06%)
DEHP1	52744476	51304521(97.27%)	3925037(7.44%)	47379484(89.83%)
DEHP2	42591388	41375119(97.14%)	3312422(7.78%)	38062697(89.37%)
DEHP3	45408678	44149539(97.23%)	4410572(9.71%)	39738967(87.51%)
MPs_DEHP1	45738822	44344832(96.95%)	3611841(7.9%)	40732991(89.06%)
MPs_DEHP2	48841006	47588848(97.44%)	4835761(9.9%)	42753087(87.54%)
MPs_DEHP3	44437298	43394032(97.65%)	3752024(8.44%)	39642008(89.21%)

Table S5. Statistical table of the number of differential genes.

Diff_group	Total DEG	Up	Down
DEHP_vs_Con	2330	1218	1112
DEHP_vs_MPs	1717	988	729
MPs_DEHP_vs_Con	1844	1039	805
MPs_DEHP_vs_DEHP	30	19	11
MPs_DEHP_vs_MPs	1974	1057	917
MPs_vs_Con	176	74	102
